# Neurodevelopment of HIV-exposed uninfected children in South Africa: outcomes from an observational birth cohort study

**DOI:** 10.1016/S2352-4642(19)30250-0

**Published:** 2019-11

**Authors:** Catherine J Wedderburn, Shunmay Yeung, Andrea M Rehman, Jacob A M Stadler, Raymond T Nhapi, Whitney Barnett, Landon Myer, Diana M Gibb, Heather J Zar, Dan J Stein, Kirsten A Donald

**Affiliations:** a Department of Paediatrics and Child Health, Red Cross War Memorial Children's Hospital, University of Cape Town, Cape Town, South Africa; b South African Medical Research Council Unit on Child and Adolescent Health, University of Cape Town, Cape Town, South Africa; c Division of Epidemiology and Biostatistics, School of Public Health and Family Medicine, University of Cape Town, Cape Town, South Africa; d Centre for Infectious Diseases Epidemiology and Research, School of Public Health and Family Medicine, University of Cape Town, Cape Town, South Africa; e Department of Psychiatry and Mental Health, University of Cape Town, Cape Town, South Africa; f South African Medical Research Council Unit on Risk and Resilience in Mental Disorders, University of Cape Town, Cape Town, South Africa; g Department of Clinical Research, London School of Hygiene & Tropical Medicine, London, UK; h Medical Research Council Tropical Epidemiology Group, London School of Hygiene & Tropical Medicine, London, UK; i Medical Research Council Clinical Trials Unit, University College London, London, UK

## Abstract

**Background:**

HIV infection is known to cause developmental delay, but the effects of HIV exposure without infection during pregnancy on child development are unclear. We compared the neurodevelopmental outcomes of HIV-exposed uninfected and HIV-unexposed children during their first 2 years of life.

**Methods:**

Pregnant women (>18 years of age) at 20–28 weeks' gestation were enrolled into the Drakenstein Child Health cohort study while attending routine antenatal appointments at one of two peri-urban community-based clinics in Paarl, South Africa. Livebirths born to enrolled women during follow-up were included in the birth cohort. Mothers and infants received antenatal and postnatal HIV testing and antiretroviral therapy per local guidelines. Developmental assessments on the Bayley Scales of Infant and Toddler Development, third edition (BSID-III), were done in a subgroup of infants at 6 months of age, and in the full cohort at 24 months of age, with assessors masked to HIV exposure status. Mean raw scores and the proportions of children categorised as having a delay (scores <–2 SDs from the reference mean) on BSID-III were compared between HIV-exposed uninfected and HIV-unexposed children.

**Findings:**

1225 women were enrolled between March 5, 2012, and March 31, 2015. Of 1143 livebirths, 1065 (93%) children were in follow-up at 6 months and 1000 (87%) at 24 months. Two children were diagnosed with HIV infection between birth and 24-month follow-up and were excluded from the analysis. BSID-III assessments were done in 260 (24%) randomly selected children (61 HIV-exposed uninfected, 199 HIV-unexposed) at 6 months and in 732 (73%) children (168 HIV-exposed uninfected, 564 HIV-unexposed) at 24 months. All HIV-exposed uninfected children were exposed to antiretrovirals (88% to maternal triple antiretroviral therapy). BSID-III outcomes did not significantly differ between HIV-exposed uninfected and HIV-unexposed children at 6 months. At 24 months, HIV-exposed uninfected children scored lower than HIV-unexposed for receptive language (adjusted mean difference −1·03 [95% CI −1·69 to −0·37]) and expressive language (−1·17 [–2·09 to −0·24]), whereas adjusted differences in cognitive (−0·45 [–1·32 to 0·43]), fine motor (0·09 [–0·49 to 0·66]), and gross motor (−0·41 [–1·09 to 0·27]) domain scores between groups were not significant. Correspondingly, the proportions of HIV-exposed uninfected children with developmental delay were higher than those of HIV-unexposed children for receptive language (adjusted odds ratio 1·96 [95% CI 1·09 to 3·52]) and expressive language (2·14 [1·11 to 4·15]).

**Interpretation:**

Uninfected children exposed to maternal HIV infection and antiretroviral therapy have increased odds of receptive and expressive language delays at 2 years of age. Further long-term work is needed to understand developmental outcomes of HIV-exposed uninfected children, especially in regions such as sub-Saharan Africa that have a high prevalence of HIV exposure among children.

**Funding:**

Bill & Melinda Gates Foundation, SA Medical Research Council, Wellcome Trust.

## Introduction

More than 1·4 million children are born to HIV-infected mothers annually, and 90% live in sub-Saharan Africa.^1^ However, following the success of programmes for the prevention of mother-to-child transmission of HIV through maternal antiretroviral therapy (ART), most children born to HIV-infected mothers are not infected with HIV, and there are an estimated 14·8 million HIV-exposed uninfected children worldwide.^1^ Whereas paediatric HIV infection is known to delay neurodevelopment,^2^ the outcomes of HIV-exposed uninfected children are less clear.

HIV-exposed uninfected children have increased morbidity and mortality,^3^ and might also have adverse developmental outcomes compared with HIV-unexposed children. Several studies have described varying degrees of impaired cognitive, language, and motor development in HIV-exposed uninfected children, particularly in low-resource settings,^4^, ^5^, ^6^ including South Africa.^7^ However, other studies have found no substantial evidence of developmental delay.^8^, ^9^ Few studies have investigated children exposed to current first-line antiretroviral drug therapy in sub-Saharan Africa, and most have not documented infant feeding mode, which has been associated with neurodevelopment.^7^ Additionally, many previous studies have had small sample sizes or cross-sectional design, or have lacked adequate HIV-unexposed comparison groups or assessment of potential confounders. Given the heterogeneity of studies to date, uncertainty remains regarding the developmental outcomes of HIV-exposed uninfected children.

**Research in context**

**Evidence before this study**

We searched six external databases (MEDLINE, PubMed, Embase, PsychINFO, Africa-Wide Information, and Global Health) for articles published from database inception until April 30, 2019, that examined the neurodevelopment of HIV-exposed uninfected children. The search terms used included those related to the concepts of “child”, “neurodevelopment”, and “HIV / antiretroviral (ARV) drugs”, which were adapted for use with the different databases and combined with database-specific filters where available. We excluded studies published before the year 2000. Longitudinal studies from high HIV-burden countries in the current era of antiretroviral therapy (ART) are scarce, as highlighted by reviews. Many previous studies have had small sample sizes and lacked adequate comparison groups or assessment of potential confounders. Overall, there is growing recognition that HIV-exposed uninfected children might have poorer developmental outcomes compared with HIV-unexposed children, particularly in low-income and middle-income countries. Several studies have reported impairments in cognitive, language, or motor function. Other studies, including one from Uganda and Malawi, did not find substantial differences between HIV-exposed uninfected and HIV-unexposed children. Given that most studies have been cross-sectional and that not all data are consistent, the exact nature of developmental delay and the clinical relevance remain unclear.

**Added value of this study**

This population-based study investigates the effect of HIV and antiretroviral exposure on early child development in a well characterised South African cohort in the current ART era. We found no difference between HIV-exposed uninfected and HIV-unexposed children from the same environment at 6 months in any developmental domain in our cohort. However, by 24 months, HIV-exposed uninfected children had significantly poorer receptive and expressive language outcomes and had increased risks of delay (scores <–2 SD from the reference mean of Bayley Scales of Infant and Toddler Development, third edition) in these domains compared with HIV-unexposed children after controlling for relevant confounders. Additionally, in an exploratory analysis, we found an association between language delay and maternal immunosuppression. Cognitive and motor outcomes at 24 months were not significantly affected by HIV exposure.

**Implications of all the available evidence**

HIV-exposed uninfected children might be at higher risk of delayed language development (both receptive and expressive) compared with HIV-unexposed children, and these delays are identifiable as early as 24 months of age. Consistently, previous studies from both high-resource and low-resource settings have suggested an association between HIV exposure without infection and adverse language outcomes. The increased severe language delay is concerning, and further follow-up of these children is needed to ascertain whether the effects have a continued impact later in life and to delineate the potential mechanisms. Identifying those children who are most susceptible to poor developmental outcomes is necessary to focus interventions and improve child health outcomes. Given that almost a quarter of South African children are exposed to HIV, and that numbers of HIV-exposed uninfected children are increasing globally, these findings could have important implications for public health policies.

Neurodevelopment during fetal and early life, a time of intense brain maturation, forms the basis of academic achievement and economic productivity. Identifying the children most susceptible to delays in neurodevelopment is necessary to focus interventions and improve child health outcomes. According to proxy measures of stunting and poverty, sub-Saharan Africa has the highest proportion of children at risk of not reaching their developmental potential worldwide.^10^ Therefore, understanding whether neurodevelopment is impaired in the expanding population of HIV-exposed uninfected children in this region is important, and studies are needed to ascertain the exact nature of any developmental delay with appropriate controls in the current ART era.^3^

We aimed to compare neurodevelopmental outcomes of HIV-exposed uninfected and HIV-unexposed children from the South African Drakenstein Child Health Study (DCHS) during their first 2 years of life.

## Methods

### Study design and participants

The DCHS is a population-based birth cohort study based in Paarl (a peri-urban area of the Western Cape, South Africa), investigating the early-life determinants of child health and development.^11^, ^12^ The antenatal HIV prevalence in this study population is 21%. Pregnant women who were older than 18 years of age, receiving antenatal care at a participating site, and intending to reside in the area for at least a year were enrolled into the study at 20–28 weeks' gestation while attending routine antenatal appointments.^11^, ^12^ Participants were recruited from two community-based clinics: T C Newman clinic (serving a mixed-ancestry community who speak Afrikaans) and Mbekweni clinic (serving a predominantly black African community who speak isiXhosa), both of which provide free maternal and child care. Written informed consent was obtained annually for mother–child pairs. A subgroup of randomly selected children underwent developmental assessment at 6 months, and all available children were assessed at age 24 months.

This study was approved by the human research ethics committee of the Faculty of Health Sciences, University of Cape Town (approval numbers 401/2009 and 044/2017), and by the London School of Hygiene & Tropical Medicine observational and interventions research ethics committee (approval number 11903).

### Procedures

Maternal HIV status during pregnancy was confirmed by routine HIV testing at booking, with retesting of HIV-negative mothers every 12 weeks, in accordance with Western Cape prevention of mother-to-child transmission of HIV guidelines. Additionally, maternal interviews and HIV status reviews of mothers and children were done by study staff at the child's birth, age 6 weeks, and every 6 months thereafter. All HIV-infected mothers were initiated on antiretroviral drugs according to prevention of mother-to-child transmission guidelines at the time: three-drug ART (the first-line ART regimen consisted of two nucleoside reverse transcriptase inhibitors plus a non-nucleoside reverse transcriptase inhibitor, commonly tenofovir plus emtricitabine plus efavirenz) or zidovudine from 14 weeks' gestation, depending on maternal clinical and immunological status (before May, 2013); or ART for life for all pregnant women (from May, 2013). All HIV-exposed uninfected children received prophylaxis (nevirapine alone or combined with zidovudine) from birth. HIV and ART data were collected by triangulating clinic and hospital folder information and maternal self-report interviews. Maternal CD4 cell count and viral load data were obtained from the online National Health Laboratory Service system. Where there were multiple results, the highest viral load during pregnancy was taken, and the lowest CD4 cell count within 1 year before childbirth and 3 months post-birth was used to maximise numbers. All HIV-exposed children received HIV testing as per local guidelines. HIV detection was done by PCR at age 6 weeks, and by rapid antibody, PCR, or ELISA at age 9 months and 18 months. HIV-exposed uninfected children were confirmed to have a negative HIV test result at age 18 months or a negative test after cessation of breastfeeding if this occurred at more than 18 months of age. HIV-unexposed children were defined as children born to mothers without HIV infection.

Sociodemographic data were collected between weeks 28 and 32 of gestation by trained study staff using structured interviews and questionnaires adapted from the South African Stress and Health study.^11^, ^12^

Detailed birth data were obtained at delivery. Gestational age was calculated using the best estimated delivery date based on antenatal ultrasound, the last menstrual period, or the symphysis-fundal height. Prematurity was defined as birth at less than 37 weeks' gestation. Infant feeding method and exclusive breastfeeding duration were documented by maternal report at age 6–14 weeks, 6 months, and 9 months.

Maternal psychosocial data were collected antenatally between weeks 28 and 32 of gestation.^12^ Maternal alcohol use during pregnancy was assessed using the Alcohol, Smoking, and Substance Involvement Screening Test.^12^ Material tobacco exposure during pregnancy was assessed by use of the IMMULITE 1000 nicotine metabolite kit (Siemens Medical Solutions Diagnostics, Glyn Rhonwy, UK) to measure antenatal urine cotinine concentration. Maternal depression was assessed with the Edinburgh Postnatal Depression Scale (with a score of ≥13 considered to indicate depression).^12^

### Developmental assessment

Neurodevelopment was measured with the Bayley Scales of Infant and Toddler Development, third edition (BSID-III),^13^, ^14^ which is widely used internationally and has been validated in South Africa with reported values similar to those of the BSID-III US-based reference population.^15^, ^16^ The objectively measured assessment consists of five subscales: cognition, receptive language, expressive language, fine motor, and gross motor. Trained assessors masked to HIV exposure status alternated testing between clinics, assessing equal numbers of children at each site and offering language prompts in the child's preferred language. Any child with significant developmental delay was referred to the relevant health-care service. Inter-assessor reliability was assured through training and supervision. Assessors were monitored by a paediatric neurodevelopmental specialist throughout, who periodically observed assessments to ensure standardised data collection across sites, accuracy, and continued agreement between assessors. A second level of external quality control was done centrally before data capture. Scores were entered into a specialised BSID-III software programme.

260 children were randomly selected from the original cohort as a sampling frame for developmental assessment at 6 months of age. If a mother–child pair was unavailable, another child aged 6 months was invited to attend from those with study follow-up visits that week, based on child age at the time (a convenience sample). All available children were assessed at age 24 months. Mothers who were unavailable at initial contact were tried again up to three times, and children who were unwell on the day of assessment were rebooked where possible. Raw scores, scaled scores, and developmental delay are reported. Raw scores represent the sum of individual items the child passes on each subscale. Raw scores were converted to age-adjusted scaled scores standardised using normative data derived from a US reference population, with a range of 1–19 and mean of 10 (SD 3), with correction for prematurity at 6 months.^13^ A BSID-III score of less than −2 SDs from the BSID-III reference mean was used to define a clinically significant delay in any domain.^7^, ^13^

### Statistical analysis

Maternal and child sociodemographic characteristics were expressed as mean (SD) for continuous data or absolute frequencies (%) for categorical data, and were compared between HIV-exposed uninfected and HIV-unexposed children with BSID-III results at 6 months and 24 months using descriptive statistics (*t* tests or χ^2^ tests).

The associations between HIV exposure and each BSID-III subscale (cognitive, receptive language, expressive language, fine motor, and gross motor) at 6 months and 24 months of age were compared with use of linear regression models for raw scores and logistic regression models for developmental delay. Standardised effect sizes were reported as Cohen's d values. A directed acyclic graph (DAG) was constructed using DAGitty, according to previously published literature, to delineate assumptions regarding the causal pathway between HIV exposure and neurodevelopment. Multivariable models were then created using all potential confounders of the exposure–outcome relationship, as determined a priori by the DAG (household income, maternal education, maternal age, and sex and age of child). Residuals were checked for normality through quantile-quantile plots to confirm the linearity assumption for the models. Adjusted mean differences, adjusted odds ratios (ORs), and 95% CIs are presented.

Additional analyses were done by adjusting the models for key potential mediating variables identified from the DAG (prematurity, maternal depression, and breastfeeding [exclusive breastfeeding duration and exclusive breastfeeding to 6 months]) to assess any changes to specific domain associations. Sensitivity analyses were done to examine the effect of site and home language in a restricted subanalysis of Mbekweni clinic, where the majority of HIV-exposed uninfected children reside. A separate analysis limited to HIV-exposed uninfected children whose mothers were on first-line ART was also done. Finally, the association between maternal CD4 cell count (dichotomised into ≤500 and >500 cells per μL, as per previous guidelines,^17^ with similar group sizes) and developmental outcomes was explored in BSID-III language subscales to provide direction for future work.

Statistical analyses were done with STATA software (version 14.0). p values of less than 0·05 (two-tailed) were considered to indicate statistical significance.

### Role of the funding source

The funders had no role in the study design, data collection, data analysis, data interpretation, or writing of the report. The corresponding author had full access to all the data in the study and had final responsibility for the decision to submit for publication.

## Results

**Figure 1:**
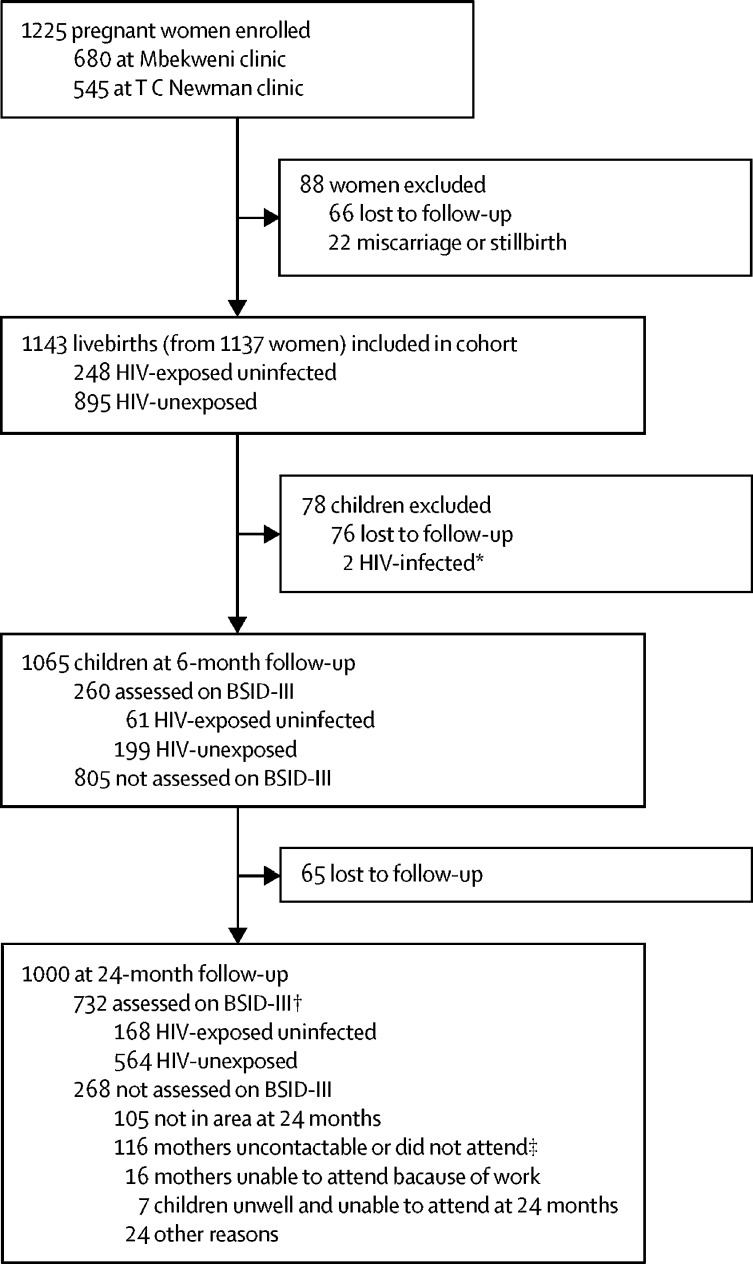
Drakenstein Child Health Study profile BSID-III=Bayley Scales of Infant and Toddler Development, third edition. *Excluded from this analysis, but not from the Drankenstein Child Health Study follow-up. †209 (80%) of the 260 children assessed on BSID-III at 6 months were also assessed at 24 months (46 HIV-exposed uninfected and 163 HIV-unexposed). ‡No show because of violence, poor weather conditions, or seasonal work.

Between March 5, 2012, and March 31, 2015, 1225 pregnant women were enrolled into the study. Of 1143 live infants born to 1137 enrolled women, follow-up was available for 1065 (93%) children at 6 months and 1000 (87%) at 24 months of age, excluding two children with HIV infection identified between birth and 24-month follow-up (figure 1). BSID-III assessments were done for a subgroup of 260 (24%) uninfected children (61 HIV-exposed uninfected and 199 HIV-unexposed) at 6 months and for 732 (73%) children (168 HIV-exposed uninfected and 564 HIV-unexposed) at 24 months. Demographic characteristics of HIV-exposed uninfected and HIV-unexposed children at 6 months and 24 months were similar (table 1). Mothers with and mothers without HIV infection did not differ significantly in terms of household income, employment status, marital status, maternal depression, or alcohol use during pregnancy. Similar proportions of HIV-exposed uninfected and HIV-unexposed children were born premature or with low birthweight. Most children attended the BSID-III assessment with their mother. Exclusive breastfeeding was uncommon, with less than 20% of mothers exclusively breastfeeding for 6 months. Among children who exclusively breastfed, breastfeeding duration was shorter for HIV-exposed uninfected than for HIV-unexposed children. Overall, a higher proportion of children seen at the Mbekweni clinic were HIV-exposed uninfected than were children seen at the T C Newman clinic. HIV-infected mothers were on average older at the time of childbirth than were uninfected mothers.

**Table 1:** Demographic characteristics of children assessed on BSID-III at 6 months and 24 months according to HIV exposure

		**6 months**			**24 months**		
		HIV-exposed (N=61)	HIV-unexposed (N=199)	p value	HIV-exposed (N=168)	HIV-unexposed (N=564)	p value
Child age at BSID-III assessment, days		184 (11)	184 (12)	0·84	732 (16)	733 (16)	0·53
Sex		..	..	0·70	..	..	0·20
	Male	33/61 (54%)	102/199 (51%)	..	94/168 (56%)	284/564 (50%)	..
	Female	28/61 (46%)	97/199 (49%)	..	74/168 (44%)	280/564 (50%)	..
Site (proxy for primary language)		..	..	<0·0001	..	..	<0·0001
	T C Newman clinic (Afrikaans)	3/61 (5%)	130/199 (65%)		13/168 (8%)	331/564 (59%)	..
	Mbekweni clinic (isiXhosa)	58/61 (95%)	69/199 (35%)		155/168 (92%)	233/564 (41%)	..
Monthly household income, South African rand*		..	..	0·74	..	..	0·93
	<1000	26/61 (43%)	88/199 (44%)	..	66/168 (39%)	221/564 (39%)	..
	1000–5000	30/61 (49%)	89/199 (45%)	..	83/168 (49%)	273/564 (48%)	..
	>5000	5/61 (8%)	22/199 (11%)	..	19/168 (11%)	70/564 (12%)	..
Maternal education		..	..	0·99	..	..	0·0010
	Any primary	4/61 (7%)	11/199 (6%)	..	19/168 (11%)	40/564 (7%)	..
	Any secondary	34/61 (56%)	115/199 (58%)	..	108/168 (64%)	290/564 (51%)	..
	Completed secondary	20/61 (33%)	64/199 (32%)	..	37/168 (22%)	196/564 (35%)	..
	Any tertiary	3/61 (5%)	9/199 (5%)	..	4/168 (2%)	38/564 (7%)	..
Maternal death		0	0	..	2/168 (1%)	1/564 (0·2%)	0·071
Mother attended BSID-III assessment		54/60 (90%)	179/198 (90%)	0·93	143/168 (85%)	470/549 (86%)	0·87
Mother in employment		15/61 (25%)	43/199 (22%)	0·63	41/168 (24%)	142/564 (25%)	0·84
Mother married or cohabitating		24/61 (39%)	71/198 (36%)	0·62	75/168 (45%)	217/563 (39%)	0·16
Maternal age at birth, years		29·8 (5·4)	25·5 (5·3)	<0·0001	30·4 (5·3)	26·3 (5·6)	<0·0001
Gestational age at delivery, weeks		38·8 (2·3)	38·7 (2·2)	0·80	38·5 (2·6)	38·6 (2·5), N=562	0·64
Premature birth (<37 weeks' gestation)		9/61 (15%)	24//199 (12%)	0·58	23/168 (14%)	81/562 (14%)	0·81
Birthweight							
	Mean (SD), g	3102 (501)	3043 (552)	0·46	3022 (583)	3039 (579)	0·74
	Low birthweight (<2·5 kg)	7/61 (11%)	25/199 (13%)	0·82	23/168 (14%)	84/564 (15%)	0·70
Birth length, cm		49·5 (4·3)	49·8 (3·7), N=198	0·60	49·9 (4·1), N=165	49·9 (3·6), N=555	0·94
Birth head circumference, cm		33·5 (1·8)	33·5 (1·9), N=198	0·88	33·6 (2·1), N=167	33·5 (2·1), N=558	0·72
WHO length/height-for-age Z-score		−0·44 (1·5), N=59	−0·44 (1·7), N=189	0·98	−1·17 (1·2), N=130	−1·09 (1·2), N=449	0·49
Maternal smoking during pregnancy (urine cotinine concentration)†		..	..	0·010	..	..	0·10
	Active (≥500 ng/mL)	14/61 (23%)	87/195 (45%)	..	46/164 (28%)	204/550 (37%)	..
	Passive (10–500 ng/mL)	31/61 (51%)	71/195 (36%)	..	79/164 (48%)	229/550 (42%)	..
	Non-smoker (<10 ng/mL)	16/61 (26%)	37/195 (19%)	..	39/164 (24%)	117/550 (21%)	..
Moderate-to-severe maternal alcohol use during pregnancy		8/55 (15%)	44/192 (23%)	0·18	16/148 (11%)	79/507 (16%)	0·15
Maternal depression during pregnancy		9/55 (16%)	57/191 (30%)	0·047	33/149 (22%)	123/508 (24%)	0·60
Exclusive breastfeeding duration, months							
	Mean (SD)	1·1 (2·0)	2·4 (2·0)	<0·0001	1·5 (2·1)	2·3 (1·9), N=563	<0·0001
	6 months‡	7/61 (11%)	37/199 (19%)	0·20	24/168 (14%)	100/563 (18%)	0·29
Maternal HIV diagnosis timepoint							
	Before pregnancy	44/59 (75%)	..	..	122/163 (75%)	..	..
	During pregnancy	15/59 (25%)	..	..	41/163 (25%)	..	..
Maternal CD4 cell count in pregnancy, cells/μL							
	Median (range)	522 (298–691), N=56	..	..	441 (294–618), N=151	..	..
	<200	6/56 (11%)	..	..	17/151 (11%)	..	..
	200–350	12/56 (21%)	..	..	37/151 (25%)	..	..
	350–500	9/56 (16%)	..	..	33/151 (22%)	..	..
	>500	29/56 (52%)	..	..	64/151 (42%)	..	..
Highest maternal viral load during pregnancy							
	Below detectable limit (<40 copies/mL)	25/36 (69%)	..	..	69/108 (64%)	..	..
	Detectable (≥40–1000 copies/mL)	6/36 (17%)	..	..	25/108 (23%)	..	..
	Unsuppressed (>1000 copies/mL)	5/36 (14%)	..	..	14/108 (13%)	..	..
Antiretroviral drug initiation							
	Before pregnancy	22/59 (37%)	..	..	71/165 (43%)	..	..
	During pregnancy	37/59 (63%)	..	..	94/165 (57%)	..	..
Antiretroviral regimen during pregnancy							
	Prevention of mother-to-child transmission prophylaxis (zidovudine)	9/58 (16%)	..	..	20/163 (12%)	..	..
	First-line triple therapy§	48/58 (83%)	..	..	132/163 (81%)	..	..
	Second-line or third-line therapy	1/58 (2%)	..	..	11/163 (7%)	..	..
Infant prophylaxis							
	Nevirapine alone	55/60 (92%)	..	..	145/167 (87%)	..	..
	Nevirapine and zidovudine	5/60 (8%)	..	..	22/167 (13%)	..	..

Data are n/N (%), mean (SD), or median (IQR). Continuous variables were compared with unpaired t tests; categorical variables were compared with χ^2^ tests. All percentages calculated on non-missing values. N values are indicated where the number of participants with available data differs from the total group size. Missing data: birth head circumference (n=1 at 6 months, n=7 at 24 months); birth length (n=1 at 6 months, n=12 at 24 months); birthweight (n=1 at 24 months); WHO length/height-for-age Z score (n=12 at 6 months, n=153 at 24 months); gestation delivery (n=2 at 24 months); breastfeeding duration (n=1 at 24 months). Maternal CD4 taken as the lowest CD4 from 1 year before to 3 months after delivery to reflect maternal immunosuppression during pregnancy with the highest sample size. BSID-III=Bayley Scales of Infant and Toddler Development, third edition.

* 1000 South African rand is approximately equal to US$75.

† p=0·22 (6 months) and p=0·50 (24 months) for non-smoking versus active and passive smoking together.

‡ Exclusive breastfeeding to 6 months was defined as exclusive breastfeeding for >5 months of age.

§ A non-nucleoside reverse-transcriptase inhibitor backbone and two nucleoside reverse transcriptase inhibitors, most commonly efavirenz with tenofovir and emtricitabine as a fixed-dose combination, although some mothers received nevirapine-based treatment; of those mothers on first-line triple antiretroviral therapy, 42 (88%) at 6 months and 116 (88%) at 24 months received efavirenz-based therapy. No mothers in the HIV-unexposed group were taking any antiretrovirals during pregnancy.

Among children assessed at 24 months, HIV-infected mothers had lower educational attainment than did uninfected mothers. Median maternal CD4 was 441 cells per μL, and 69 (64%) of those with available results had an undetectable viral load. Maternal antiretroviral drug regimens included zidovudine prophylaxis for prevention of mother-to-child transmission (12%), first-line three-drug ART (81%), and second-line or third-line protease inhibitor-containing therapies (7%; appendix p 1). 43% of women initiated antiretrovirals before pregnancy, and 88% of mothers on first-line ART had efavirenz-based therapy. Children received post-exposure prophylaxis with nevirapine alone (87%), or nevirapine and zidovudine (13%).

The children with BSID-III outcomes were largely representative of the full cohort at each timepoint (appendix p 2), although at 24 months they had a higher maternal age, a higher proportion of maternal alcohol use, and a lower proportion of premature birth. The highest percentage of missing data in any single BSID-III domain was 5%.

At 6-month BSID-III assessments, HIV-exposed uninfected and HIV-unexposed children showed no significant differences in mean raw scores on any of the subscales (table 2). Mean scaled scores for all subscales were within 1 SD of the BSID-III reference mean (mean score 10 [SD 3]; appendix p 4). Numbers of children with developmental delay were low across the cohort (two to seven children in any subscale).

**Table 2:** Unadjusted and adjusted mean differences in BSID-III domain raw scores at 6 months and 24 months according to HIV exposure

	**HIV-exposed**		**HIV-unexposed**		**Unadjusted**			Adjusted*		
	N	Mean raw score (SD)	N	Mean raw score (SD)	Mean difference (95% CI)	p value	Effect size, Cohen's d (95% CI)	Mean difference (95% CI)	p value	Effect size, Cohen's d (95% CI)
**6 months**										
Cognitive	60	27·55 (2·82)	196	27·08 (3·69)	0·47 (−0·55 to 1·49)	0·37	0·13 (−0·16 to 0·42)	0·69 (−0·33 to 1·72)	0·19	0·20 (−0·09 to 0·49)
Receptive language	60	9·78 (1·55)	194	9·57 (1·65)	0·22 (−0·26 to 0·69)	0·37	0·13 (−0·16 to 0·42)	0·23 (−0·28 to 0·73)	0·38	0·14 (−0·15 to 0·43)
Expressive language	61	8·62 (2·72)	194	8·89 (2·61)	−0·26 (−1·03 to 0·50)	0·50	−0·10 (−0·39 to 0·19)	−0·42 (−1·22 to 0·39)	0·31	−0·16 (−0·45 to 0·13)
Fine motor	61	21·79 (2·85)	196	21·60 (3·03)	0·19 (−0·67 to 1·05)	0·67	0·06 (−0·22 to 0·35)	0·55 (−0·32 to 1·42)	0·22	0·19 (−0·10 to 0·47)
Gross motor	61	24·75 (3·73)	195	24·54 (3·66)	0·21 (−0·85 to 1·27)	0·70	0·06 (−0·23 to 0·34)	0·57 (−0·48 to 1·62)	0·29	0·16 (−0·13 to 0·44)
**24 months**										
Cognitive	167	54·84 (5·06)	562	55·69 (4·73)	−0·85 (−1·68 to −0·02)	0·045	−0·18 (−0·35 to −0·003)	−0·45 (−1·32 to 0·43)	0·32	−0·09 (−0·27 to 0·08)
Receptive language	165	19·83 (3·54)	556	21·10 (3·72)	−1·27 (−1·91 to −0·63)	0·0001	−0·34 (−0·51 to −0·16)	−1·03 (−1·69 to −0·37)	0·0024	−0·28 (−0·45 to −0·10)
Expressive language	158	22·91 (5·37)	542	24·45 (4·94)	−1·54 (−2·43 to −0·64)	0·0008	−0·30 (−0·48 to −0·12)	−1·17 (−2·09 to −0·24)	0·013	−0·23 (−0·41 to −0·05)
Fine motor	166	37·40 (3·34)	562	37·51 (3·10)	−0·13 (−0·68 to 0·42)	0·64	−0·04 (−0·21 to 0·13)	0·09 (−0·49 to 0·66)	0·77	0·03 (−0·15 to 0·20)
Gross motor	159	53·07 (3·37)	535	53·31 (3·66)	−0·24 (−0·88 to 0·39)	0·46	−0·07 (−0·24 to 0·11)	−0·41 (−1·09 to 0·27)	0·24	−0·11 (−0·29 to 0·06)

Residuals were assessed for each model using quantile–quantile plots and were normally distributed. Negative mean difference estimates indicate that HIV exposure was associated with lower total raw scores in that BSID-III domain (ie, poorer outcomes). BSID-III=Bayley Scales of Infant and Toddler Development, third edition.

* Adjusted for child age, child sex, maternal education, household income, and maternal age.

At 24 months, univariate analysis of BSID-III cognition outcomes showed that HIV-exposed uninfected children had lower mean raw scores than did HIV-unexposed children (mean difference −0·85 [95% CI −1·68 to −0·02]). However, this difference was attenuated after adjustment for confounding variables (−0·45 [–1·32 to 0·43]; table 2). The proportions of children with developmental delay (defined as a scaled score less than −2 SDs from the BSID-III reference mean) on the cognition subscale were similar between HIV-exposed uninfected and HIV-unexposed children (11% *vs* 9%, adjusted OR 1·01 [95% CI 0·55 to 1·85]; table 3).

**Table 3:** Odds of developmental delay by BSID-III domain at 24 months according to HIV exposure

	**Infants with delay, n/N (%)**		**Unadjusted**		Adjusted*	
	HIV-exposed	HIV-unexposed	OR (95% CI)	p value	OR (95% CI)	p value
Cognitive	18/167 (11%)	52/562 (9%)	1·18 (0·67 to 2·09)	0·56	1·01 (0·55 to 1·85)	0·97
Receptive language	23/165 (14%)	40/556 (7%)	2·09 (1·21 to 3·61)	0·0081	1·96 (1·09 to 3·52)	0·025
Expressive language	18/158 (11%)	31/542 (6%)	2·12 (1·15 to 3·90)	0·016	2·14 (1·11 to 4·15)	0·024
Fine motor	6/166 (4%)	12/562 (2%)	1·72 (0·64 to 4·65)	0·29	1·53 (0·53 to 4·42)	0·44
Gross motor	6/159 (4%)	19/535 (4%)	1·07 (0·42 to 2·71)	0·90	1·23 (0·44 to 3·43)	0·69

We did a complete case analysis by outcome. ORs greater than 1 indicate that HIV exposure was associated with higher risk of delay in that BSID-III domain (ie, poorer outcomes). Total number of participants assessed (N) for each domain was the same for both unadjusted and adjusted models; no covariates had missing data. BSID-III=Bayley Scales of Infant and Toddler Development, third edition. OR=odds ratio.

* Adjusted for child age, child sex, maternal education, household income, and maternal age.

In both unadjusted and adjusted models, HIV exposure was associated with lower 24-month scores for receptive language (adjusted mean difference −1·03 [–1·69 to −0·37]) and expressive language (adjusted mean difference −1·17 [–2·09 to −0·24]; table 2). Although the effect sizes were small (adjusted Cohen's d −0·28 [95% CI −0·45 to −0·10] for receptive and −0·23 [–0·41 to −0·05] for expressive language), these differences represent reduced ability to understand the meaning of words and commands in receptive language, and reduced ability to use sounds and words to communicate in expressive language. Correspondingly, greater proportions of HIV-exposed uninfected than HIV-unexposed children had delayed development in receptive language (14% *vs* 7%, adjusted OR 1·96 [1·09 to 3·52]) and expressive language (11% *vs* 6%, 2·14 [1·11 to 4·15]; table 3).

There were no differences in mean raw scores or the proportions of children with developmental delays between HIV-exposed uninfected and HIV-unexposed children with regard to fine motor or gross motor domains on 24-month BSID-III assessment (Table 2, Table 3).

**Figure 2:**
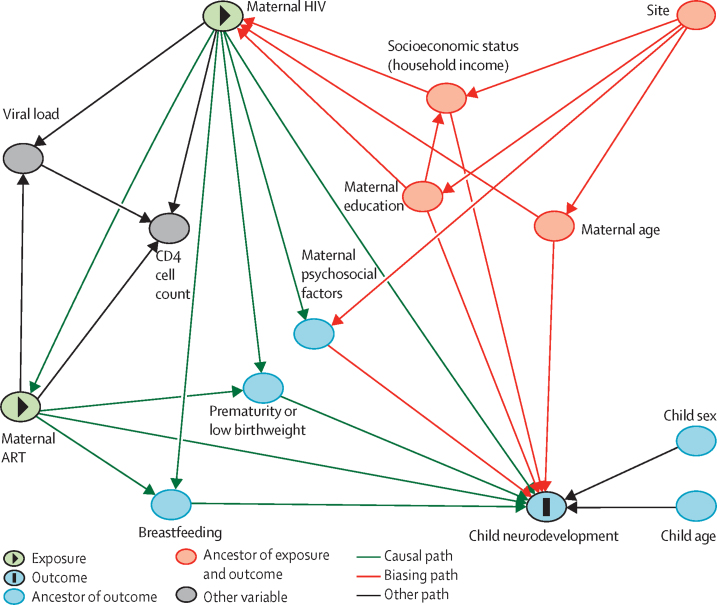
Directed acyclic graph We constructed a directed acyclic graph using DAGitty to examine for possible confounding in the relationship between HIV and ART exposure and child developmental performance on the Bayley Scales of Infant and Toddler Development, third edition, at 6 months and 24 months in the Drakenstein Child Health Study, using multiple sources.^3^, ^10^, ^11^, ^12^ In this model, site acts as a proxy for home language and ethnicity. Maternal psychosocial factors include maternal depression, alcohol use, and smoking. Minimal sufficient adjustment sets for estimating the total effect of maternal HIV and ART exposure on child neurodevelopment include socioeconomic status (household income), maternal education, and maternal age. ART=antiretroviral therapy.

In sensitivity analyses adjusting separately for exclusive breastfeeding, premature birth, and maternal depression (factors identified to potentially be on the causal pathway between HIV exposure and neurodevelopment; figure 2, appendix pp 5–7), HIV exposure remained associated with lower receptive and expressive language raw scores. Two exclusive breastfeeding classifications were examined (exclusive breastfeeding duration and exclusive breastfeeding to 6 months [defined as exclusive breastfeeding for >5 months of age]) and, in both cases, the exposure–outcome relationship remained the same. To address between-group differences in site and home language, we did a restricted analysis of children from Mbekweni clinic (appendix p 8). The overall trends in associations were similar to those of the full sample, the point estimates held, and the proportion of children with delayed development in receptive language and expressive language remained the same, although precision was reduced (which was expected with the smaller sample size). Alcohol exposure (appendix p 9) showed no effect on the associations between receptive or expressive language development and HIV exposure.

In a subgroup analysis comparing only HIV-exposed uninfected children whose mothers were initiated on first-line triple ART (n=132) with HIV-unexposed children, language impairments remained associated with HIV exposure (appendix p 10). In an exploratory analysis comparing outcomes among children born to mothers with and without immunosuppression, a maternal CD4 cell count of 500 cells per μL or less was associated with lower receptive and expressive language scores and increased prevalence of developmental delays in these domains versus children born to mothers without HIV infection (table 4).

**Table 4:** Receptive and expressive language outcomes on BSID-III at 24 months according to maternal CD4 cell counts

	**N**	**Raw scores**					**Delayed development**				
		Mean (SD)	Unadjusted		Adjusted*		Delayed, N (%)	Unadjusted		Adjusted*	
			Mean difference (95% CI)	p value	Mean difference (95% CI)	p value		OR (95% CI)	p value	OR (95% CI)	p value
**Receptive language**											
HIV-unexposed	556	21·10 (3·72)	0·00 (ref)	..	0·00 (ref)	..	40 (7%)	1·00 (ref)	..	1·00 (ref)	..
Maternal CD4 count >500 cells per μL	62	20·35 (3·24)	−0·74 (−1·71 to 0·22)	0·13	−0·48 (−1·45 to 0·49)	0·33	7 (11%)	1·64 (0·70 to 3·84)	0·25	1·56 (0·65 to 3·73)	0·32
Maternal CD4 count ≤500 cells per μL	86	19·27 (3·70)	−1·83 (−2·67 to −0·99)	<0·0001	−1·54 (−2·40 to −0·68)	0·0005	14 (16%)	2·51 (1·30 to 4·84)	0·0061	2·40 (1·19 to 4·83)	0·014
**Expressive language**											
HIV-unexposed	542	24·45 (4·94)	0·00 (ref)	..	0·00 (ref)	..	31 (6%)	1·00 (ref)	..	1·00 (ref)	..
Maternal CD4 count >500 cells per μL	60	23·87 (5·04)	−0·58 (−1·92 to 0·76)	0·40	−0·16 (−1·50 to 1·18)	0·82	5 (8%)	1·50 (0·56 to 4·01)	0·42	1·42 (0·52 to 3·89)	0·50
Maternal CD4 count ≤500 cells per μL	81	22·09 (5·47)	−2·36 (−3·53 to −1·19)	<0·0001	−1·92 (−3·12 to −0·72)	0·0018	11 (14%)	2·59 (1·25 to 5·38)	0·011	2·73 (1·24 to 6·03)	0·013

ORs greater than 1 indicate higher risk of delay in that BSID-III domain (ie, poorer outcomes) versus the reference group; negative mean difference estimates indicate lower total raw scores in that domain (ie, poorer outcomes) versus the reference group. Maternal CD4 taken as the lowest CD4 from 1 year before to 3 months after delivery to reflect maternal immunosuppression during pregnancy with the highest sample size. OR=odds ratio. BSID-III=Bayley Scales of Infant and Toddler Development, third edition.

* Adjusted for child age, child sex, maternal education, household income, and maternal age.

## Discussion

The results from this South African birth cohort show that HIV-exposed uninfected children had poorer language outcomes at 24 months, but not at 6 months, when compared with HIV-unexposed children. To our knowledge, this is the largest longitudinal study to report delayed language development in both receptive and expressive domains in HIV-exposed uninfected children in South Africa, building on previous literature^18^, ^19^, ^20^, ^21^ suggesting that language might be impaired in this population.

Overall, children in this cohort showed increased developmental impairment over time. At 6 months, developmental performance was no different between HIV-exposed uninfected and HIV-unexposed children. More subtle language impairments might not easily be identified at such a young age before explicit verbal communication has developed. Alcock and colleagues also found worsening language outcomes in older (aged 16–30 months *vs* 8–15 months) HIV-exposed uninfected children in Kenya.^18^ In our study, by 24 months of age, HIV-exposed uninfected children had language impairment in terms of both raw scores and formal delay categorisation, suggesting clinically significant impairment. Although the effect sizes were small, these findings are concerning in the context of a growing HIV-exposed uninfected population in sub-Saharan Africa. The associations between exposure to HIV and antiretroviral therapy and poorer language outcomes remained after examining infant feeding method, maternal depression, and prematurity as potential mediators. We did not find any associations between exposure to HIV and antiretroviral therapy and cognitive or motor development, similar to the results of studies from Botswana, Uganda, and Malawi.^8^, ^9^ However, the BSID-III might underestimate delay, which could also contribute to the lack of differences.^16^

Previous research has found language to be particularly affected in HIV-infected children, and has suggested that language development is also possibly affected in HIV-exposed uninfected children in both high-resource and low-resource settings, supporting our results.^2^, ^18^ A study of HIV-exposed uninfected children in the USA found increased risk of language impairments compared with population norms.^19^ In the Democratic Republic of the Congo, expressive language (and motor) delays were found in HIV-uninfected preschool children born to mothers with HIV/AIDS.^20^ A study in Botswana reported that HIV-exposed uninfected 2-year olds had increased adverse expressive language outcomes,^9^ and receptive language was impaired in HIV-exposed uninfected children aged 3 years in Uganda.^21^ A recent South African study of 1-year-old children did not find any language delay; however, only expressive language was assessed.^7^ It is possible that early exposures only manifest later and that language delay might not be evident as early as 12 months of age, as observed in the current study.

We assessed language using the BSID-III, which measures preverbal communication, vocabulary development, and recognition of common objects and animals. The tasks assessed in children younger than 24 months are more universally applicable than those for older children. The BSID-III has been validated in South Africa in infancy and found to be a culturally appropriate tool.^15^, ^16^ In young children (up to 2 years of age), research has shown many similarities in the sequence of language acquisition and vocabulary growth across languages and communities.^22^ Despite site differences, the HIV-exposed uninfected and HIV-unexposed groups in the current study had similar socioeconomic backgrounds, and the results of the analysis restricted to children seen at the Mbekweni clinic (isiXhosa-speaking) with nearly 400 children support the main findings (appendix p 8).

The mechanisms for the observed developmental delays require further investigation to understand whether a particular subgroup of HIV-exposed uninfected children might be most susceptible. Our exploratory analyses suggest an effect of maternal immunosuppression on neurodevelopment, with maternal CD4 cell counts of 500 cells per μL or fewer associated with poorer language outcomes than those of unexposed children. Growing evidence indicates that maternal immune activation affects neurodevelopment in utero^23^ and neuroinflammation might have an important role in cognitive dysfunction.^24^ Previously reported neuroimaging findings from a neonatal subgroup of the DCHS found white matter differences between HIV-exposed uninfected and HIV-unexposed neonates.^25^ These CD4 results from our study also support findings from a previous study that showed that maternal viral load might predict poorer developmental outcomes,^8^ highlighting the importance of optimising maternal health for child neurodevelopment.

Multiple socioeconomic, environmental, and biological factors influence child neurodevelopment.^10^ We adjusted for potential confounders in our analyses; however, the effects of HIV exposure on language development might be mediated through other pathways, including parent–child interaction, that are affected by caregiver physical or psychological health, for which further investigation is needed to inform interventions. Evidence for other possible mechanisms, including antiretroviral neurotoxicity, is lacking, and deciphering the role (if any) of antiretroviral neurotoxicity from HIV exposure is challenging. Antiretrovirals are known to cross the placental barrier and have been linked to mitochondrial toxicity, preterm birth, and biological deficits.^26^ In a US cohort study, language outcomes were affected by specific antiretrovirals (including atazanavir associated with late language emergence at 1 year of age and tenofovir with speech impairment at 3 years but not 5 years of age).^19^ In Botswana, no effect of monotherapy versus triple ART therapy on developmental outcomes was observed,^27^ although efavirenz exposure was associated with lower receptive language scores at 2 years of age compared with non-efavirenz-containing therapies.^28^ A study from Uganda and Malawi found no increased developmental risk associated with maternal ART.^8^ Our findings were confirmed when analyses were restricted to those infants whose mothers received first-line ART (the majority of whom were receiving efavirenz-based therapy). Careful pharmacovigilance is needed going forwards to monitor potential antiretroviral neurotoxicity.

The strengths of this prospective study include the use of a validated measure of neurodevelopment, as well as the large sample size and inclusion of an appropriate control group from a similar socioeconomic environment. Additionally, the cohort recruitment spanned from 2012 to 2015, with heterogeneity in ART regimens and maternal immune status, although most mothers were on first-line ART, both of which are representative of populations across sub-Saharan Africa. The cohort had a high prevalence of sociodemographic risk factors, similar to other low-income and middle-income settings, giving this study good generalisability.^12^, ^14^ Finally, the study measured neurodevelopment at two timepoints, and follow-up continues for these children. Research in high HIV prevalence settings using longitudinal data is essential to inform global child development research priorities.^29^

This study has some important limitations. First, the sample size at 6 months was small and might have been underpowered to detect a difference at this timepoint. Second, at 24 months, not all children in the cohort received a BSID-III assessment and, although we did sensitivity analyses to explore the effect of potential bias, the possibility of selection bias remains. Third, although the BSID-III is a well recognised tool, there are reliability concerns and further standardisation in sub-Saharan African settings is required; for example, the categorisation of delay is based on scaled scores using normative US data that might not be generalisable to a South African population. However, we reported raw scores with similar patterns to the results based on delay categorisation and compared against a control group, adding validity to our outcomes. Fourth, participants might have had hearing loss that is known to affect language development, and hearing assessments are ongoing. Finally, despite similar backgrounds, there were some differences between the HIV-exposed uninfected and HIV-unexposed groups, including lower proportions of breastfed infants among HIV-exposed uninfected children. However, the prevalence of exclusive breastfeeding was low across the whole population. The multivariable models adjusted for confounders and accounted for potential mediating variables, and, by restricting analyses to the Mbekweni clinic, we attempted to minimise residual confounding.

Elucidating risk factors associated with delayed development could inform effective preventive and intervention strategies. HIV exposure appears to affect emerging language development, which is foundational to school readiness and academic outcomes.^30^ As there are an estimated 14·8 million HIV-exposed uninfected children worldwide,^1^ this research could have significant public health implications. Furthermore, HIV-exposed uninfected children identified with early developmental delay might benefit from interventions to improve outcomes. Future work is needed to assess if these findings are replicated in cohorts with higher breastfeeding prevalence, and to ascertain their long-term significance. Research is also needed to define the causal pathways behind adverse outcomes in HIV-exposed uninfected children, including the role of maternal psychological and family socioeconomic factors, exposure to HIV, and the effect of ART regimens and initiation timing, to provide future interventions. Neurocognitive follow-up and neuroimaging of these children is ongoing and could add to our understanding of the mechanisms behind the observed developmental delays.^14^

In conclusion, we found uninfected children exposed to maternal HIV and antiretroviral drugs had delayed receptive and expressive language development identifiable as early as 24 months of age, compared with HIV-unexposed children from similar environments. Given that almost a quarter of children in South Africa are HIV-exposed and numbers are expanding globally, further work is needed to understand the long-term developmental outcomes of HIV-exposed uninfected children.

## References

[1] UNAIDS AIDSinfo. http://aidsinfo.unaids.org.

[2] Brackis-Cott E, Kang E, Dolezal C, Abrams EJ, Mellins CA (2009). The impact of perinatal HIV infection on older school-aged children's and adolescents' receptive language and word recognition skills. AIDS Patient Care STDs.

[3] Evans C, Jones CE, Prendergast AJ (2016). HIV-exposed, uninfected infants: new global challenges in the era of paediatric HIV elimination. Lancet Infect Dis.

[4] Le Doaré K, Bland R, Newell ML (2012). Neurodevelopment in children born to HIV-infected mothers by infection and treatment status. Pediatrics.

[5] McHenry MS, McAteer CI, Oyungu E (2018). Neurodevelopment in young children born to HIV-infected mothers: a meta-analysis. Pediatrics.

[6] Sherr L, Croome N, Parra Castaneda K, Bradshaw K (2014). A systematic review of psychological functioning of children exposed to HIV: using evidence to plan for tomorrow's HIV needs. AIDS Behav.

[7] le Roux SM, Donald KA, Brittain K (2018). Neurodevelopment of breastfed HIV-exposed uninfected and HIV-unexposed children in South Africa. AIDS.

[8] Boivin MJ, Maliwichi-Senganimalunje L, Ogwang LW (2019). Neurodevelopmental effects of ante-partum and post-partum antiretroviral exposure in HIV-exposed and uninfected children versus HIV-unexposed and uninfected children in Uganda and Malawi: a prospective cohort study. Lancet HIV.

[9] Chaudhury S, Williams PL, Mayondi GK (2017). Neurodevelopment of HIV-exposed and HIV-unexposed uninfected children at 24 months. Pediatrics.

[10] Black MM, Walker SP, Fernald LCH (2017). Early childhood development coming of age: science through the life course. Lancet.

[11] Zar HJ, Barnett W, Myer L, Stein DJ, Nicol MP (2015). Investigating the early-life determinants of illness in Africa: the Drakenstein Child Health Study. Thorax.

[12] Stein DJ, Koen N, Donald KA (2015). Investigating the psychosocial determinants of child health in Africa: the Drakenstein Child Health Study. J Neurosci Methods.

[13] Bayley N (2006). Bayley scales of infant and toddler development, 3rd edn—technical manual.

[14] Donald KA, Hoogenhout M, du Plooy CP (2018). Drakenstein Child Health Study (DCHS): investigating determinants of early child development and cognition. BMJ Paediatr Open.

[15] Ballot DE, Potterton J, Chirwa T, Hilburn N, Cooper PA (2012). Developmental outcome of very low birth weight infants in a developing country. BMC Pediatr.

[16] Rademeyer V, Jacklin L (2013). A study to evaluate the performance of black South African urban infants on the Bayley Scales of Infant Development III. S Afr J Child Health.

[17] Western Cape Government (November, 2015). The Western Cape consolidated guidelines for HIV treatment: prevention of mother-to-child transmission of HIV (PMTCT), children, adolescents and adults. https://www.westerncape.gov.za/sites/www.westerncape.gov.za/files/the_western_cape_consolidated_guidelines_for_hiv_treatment_29032016.pdf.

[18] Alcock KJ, Abubakar A, Newton CR, Holding P (2016). The effects of prenatal HIV exposure on language functioning in Kenyan children: establishing an evaluative framework. BMC Res Notes.

[19] Rice ML, Russell JS, Frederick T (2018). Risk for speech and language impairments in preschool age HIV-exposed uninfected children with in utero combination antiretroviral exposure. Pediatr Infect Dis J.

[20] Van Rie A, Mupuala A, Dow A (2008). Impact of the HIV/AIDS epidemic on the neurodevelopment of preschool-aged children in Kinshasa, Democratic Republic of the Congo. Pediatrics.

[21] Brahmbhatt H, Boivin M, Ssempijja V (2014). Neurodevelopmental benefits of antiretroviral therapy in Ugandan children aged 0–6 years with HIV. J Acquir Immune Defic Syndr.

[22] Bornstein MH, Cote LR, Maital S (2004). Cross-linguistic analysis of vocabulary in young children: Spanish, Dutch, French, Hebrew, Italian, Korean, and American English. Child Dev.

[23] Knuesel I, Chicha L, Britschgi M (2014). Maternal immune activation and abnormal brain development across CNS disorders. Nat Rev Neurol.

[24] Alford K, Vera JH (2018). Cognitive impairment in people living with HIV in the ART era: a review. Br Med Bull.

[25] Tran LT, Roos A, Fouche JP (2016). White matter microstructural integrity and neurobehavioral outcome of HIV-exposed uninfected neonates. Medicine (Baltimore).

[26] Mofenson LM (2015). Editorial commentary: new challenges in the elimination of pediatric HIV infection: the expanding population of HIV-exposed but uninfected children. Clin Infect Dis.

[27] Chaudhury S, Mayondi GK, Williams PL (2018). In-utero exposure to antiretrovirals and neurodevelopment among HIV-exposed-uninfected children in Botswana. AIDS.

[28] Cassidy AR, Williams PL, Leidner J (2019). In utero efavirenz exposure and neurodevelopmental outcomes in HIV-exposed uninfected children in Botswana. The Pediatr Infect Dis J.

[29] Dua T, Tomlinson M, Tablante E (2016). Global research priorities to accelerate early child development in the sustainable development era. Lancet Glob Health.

[30] Tamis-LeMonda C, Rodriguez E Parents' role in fostering young children's learning and language development. http://www.child-encyclopedia.com/language-development-and-literacy/according-experts/parents-role-fostering-young-childrens-learning.

