# Supplementary material for: Neurodevelopment of HIV-exposed uninfected children in South Africa: outcomes from an observational birth cohort study
Source: Lancet Child Adolesc Health. 2019 Nov;3(11):803–13. doi: 10.1016/S2352-4642(19)30250-0 (PMC6876655; doi:10.1016/S2352-4642(19)30250-0)
Supplement: Supplementary appendix [file mmc1.pdf]

THE LANCET
Child & Adolescent Health

Supplementary appendix

This appendix formed part of the original submission and has been peer reviewed. We post it as supplied by the authors.

Supplement to: Wedderburn CJ, Yeung S, Rehman AM, et al. Neurodevelopment of HIV-exposed uninfected children in South Africa: outcomes from an observational birth cohort study. *Lancet Child Adolesc Health* 2019; published online Sept 9. http://dx.doi.org/10.1016/S2352-4642(19)30250-0.

Appendix 1: Antiretroviral drug regimens received by HIV-infected mothers during pregnancy

Antiretroviral drug (ARV) regimen	BSID-III timepoint	
	**6 months HIV-infected mothers*** *N*, %	**24 months HIV-infected mothers*** *N*, %
PMTCT prophylaxis (AZT)	9/58 (16%)	20/163 (12%)
1st line three-drug antiretroviral therapy (ART)		
TDF + (3TC or FTC) + EFV (separately or fixed dose combination)	42/58 (72%)	112/163 (69%)
AZT + 3TC + EFV	-	3/163 (2%)
d4T + 3TC + EFV	-	1/163 (1%)
TDF + 3TC + NVP	4/58 (7%)	9/163 (6%)
AZT + 3TC + NVP	2/58 (3%)	6/163 (4%)
d4T + 3TC + NVP	-	1/163 (1%)
2nd/3rd line ART		
AZT + 3TC + LPV/r	1/58 (2%)	6/163 (4%)
TDF + 3TC + LPV/r	-	4/163 (2%)
AZT+ 3TC + RIT + ATV	-	1/163 (1%)

Footnotes:

Abbreviations: ART = Antiretroviral therapy; AZT = zidovudine; EFV = efavirenz; 3TC = lamivudine; FTC = emtricitabine; TDF = tenofovir; d4T = stavudine; NVP = nevirapine; LPV/r = lopinavir/ritonavir (Kaletra); RIT = ritonavir; ATV = atazanavir

* missing data at 6 months n =3; at 24 months n=5. Percentages are cited among those with non-missing values.

Appendix 2: Comparison of demographics among children who had a BSID-III assessment measured at either time-point and those who were in follow up but did not have a BSID-III.

Variables	6 months			24 months		
	BSID-III 260 (24·4%)	No BSID-III 805 (75·6%)	***P***	BSID-III 732 (73·2%)	No BSID-III 268 (26·8%)	***P***
HIV exposure	61/260 (23%)	170/805 (21%)	0.43	168/732 (23%)	51/268 (19%)	0.18
Male sex	135/260 (52%)	410/805 (51%)	0.78	378/732 (52%)	135/268 (50%)	0.72
Site (Mbekweni)	127/260 (49%)	461/805 (57%)	0.018*	388/732 (53%)	155/268 (58%)	0.17
Monthly household income (ZAR)						
< R1000 (<~$75)	114/260 (44%)	290/805 (36%)	0.056	287/732 (39%)	99/268 (37%)	0.81
R1000-R5000 (~$75-375)	119/260 (46%)	403/805 (50%)		356/732 (49%)	135/268 (50%)	
>R5000 (>~$375)	27/260 (10%)	112/805 (14%)		89/732 (12%)	34/268 (13%)	
Maternal education						
Primary	15/260 (6%)	61/805 (8%)	0.44	59/732 (8%)	13/268 (5%)	0.38
Secondary	149/260 (57%)	430/805 (53%)		398/732 (54%)	151/268 (56%)	
Completed secondary	84/260 (32%)	261/805 (32%)		233/732 (32%)	87/268 (32%)	
Any tertiary	12/260 (5%)	53/805 (7%)		42/732 (6%)	17/268 (6%)	
Maternal employment status (employed)	58/260 (22%)	228/805 (28%)	0.057	183/732 (25%)	81/268 (30%)	0.097
Relationship status (married / cohabitating)	95/259 (37%)	330/805 (41%)	0.22	292/731 (40%)	101/268 (38%)	0.52
Maternal age at birth, years, mean (SD)	26·5 (5·6)	27·1 (5·7)	0.13	27·3 (5·8)	26·3 (5·3)	0.017*
Gestational age at delivery, weeks, mean (SD)	38·7 (2·2)	38·4 (2·7)	0.17	38·6 (2·5)	38·4 (2·6)	0.25
Prematurity (<37 weeks)	33/260 (13%)	137/801 (17%)	0.092	104/730 (14%)	52/266 (20%)	0.042*
Birthweight (g), mean (SD)	3057 (540)	3029 (604)	0.50	3035 (580)	3046 (594)	0.80
Birth length (cm), mean (SD)	49.7 (3.8)	49.8 (3.8)	0.80	49.9 (3.7)	49.6 (3.7)	0.24
Birth head circumference (cm), mean (SD)	33.5 (1.9)	33.6 (2.1)	0.75	33.6 (2.1)	33.5 (2.0)	0.60
Low birthweight (<2.5kg)	32/260 (12%)	128/805 (16%)	0.16	107/732 (15%)	41/268 (15%)	0.79
Maternal smoking during pregnancy[a]						
Active	101/256 (39%)	232/772 (30%)	0.021*	250/714 (35%)	69/254 (27%)	0.067
Passive	102/256 (40%)	358/772 (46%)		308/714 (43%)	126/254 (50%)	
Non-smoker	53/256 (21%)	182/772 (24%)		156/714 (22%)	59/254 (23%)	
Maternal alcohol use during pregnancy[b]	52/247 (21%)	74/696 (11%)	<0.0001*	95/655 (15%)	21/233 (9%)	0.033*
Maternal depression[c]	66/246 (27%)	158/700 (23%)	0.18	156/657 (24%)	56/233 (24%)	0.93
Exclusive breastfeeding duration, months mean (SD)	2·1 (2·1)	2·1 (1·9)	0.96	2·2 (2·0)	2·0 (1·9)	0.22
Exclusive breastfeeding for 6 months	44/260 (17%)	123/782 (16%)	0.65	124/731 (17%)	34/261 (13%)	0.14
HIV/ARV-related variables (HEU only)						
Maternal HIV diagnosis timepoint						
Before pregnancy	44/59 (75%)	116/163 (71%)	0.62	122/163 (75%)	30/47 (64%)	0.14
During pregnancy	15/59 (25%)	47/163 (29%)		41/163 (25%)	17/47 (36%)	
Maternal CD4 cell count[d]						
Median (range) (cells/mm^3)	522 (298-691)	414 (293-575)	0.088	441 (294-618)	455 (315-635)	0.87

<350 cells/mm^3	18/56 (32%)	59/152 (39%)	0.11	54/151 (36%)	16/45 (36%)	0.999
350-500 cells/mm^3	9/56 (16%)	38/152 (25%)		33/151 (22%)	10/45 (22%)	
>500 cells/ mm^3	29/56 (52%)	55/152 (36%)		64/151 (42%)	19/45 (42%)	
Maternal Viral load (VL) in pregnancy[e]						
Lower than detectable limit (<40 copies/mL)	25/36 (69%)	64/103 (62%)	0.71	69/108 (64%)	16/25 (64%)	0.24
VL detectable (>=40-1000 copies/mL)	6/36 (17%)	23/103 (22%)		25/108 (23%)	3/25 (12%)	
Virally unsuppressed (>1000 copies/mL)	5/36 (14%)	16/103 (16%)		14/108 (13%)	6/25 (24%)	
Antiretroviral drug initiation						
Before pregnancy	22/59 (37%)	73/167 (44%)	0.39	71/165 (43%)	19/49 (39%)	0.60
During pregnancy	37/59 (63%)	94/167 (56%)		94/165 (57%)	30/49 (61%)	
Antiretroviral regimen during pregnancy						
PMTCT prophylaxis (AZT [zidovudine])	9/58 (16%)	23/165 (14%)	0.49	20/163 (12%)	10/49 (20%)	0.30
1st line Triple therapy (non-EFV)	6/58 (10%)	17/165 (10%)		16/163 (10%)	6/49 (12%)	
1st line Triple therapy (EFV-containing)	42/58 (72%)	113/165 (68%)		116/163 (71%)	32/49 (65%)	
2nd/3rd line	1/58 (2%)	12/165 (7%)		11/163 (7%)	1/49 (2%)	
Infant prophylaxis						
NVP [nevirapine] alone	55/60 (92%)	142/167 (85%)	0.19	145/167 (87%)	42/50 (84%)	0.61
NVP + AZT	5/60 (8%)	25/167 (15%)		22/167 (13%)	8/50 (16%)	

Footnotes:
Abbreviations: VL = viral load; NVP = nevirapine; AZT = zidovudine; EFV = efavirenz;

*$p<0.05$; Unpaired t-test used for continuous variables (means and SD presented); Chi-squared for categorical variables (n and % proportions presented).

Missing data at both timepoints: relationship status (n=1), maternal age at birth (n=1); gestation at delivery (n=4); smoking (n=37 at 6 months, n=32 at 24 months), Alcohol in pregnancy (n=122 at 6 months, n=112 at 24 months), depression (n=119 at 6 months, n=110 at 24 months), breast feeding (n=23 at 6 months, n=8 at 24 months); birth head circumference (n=15 at 6 months; n=14 at 24 months), birth length (n=25 at 6 months; 21 at 24 months), birthweight (n=8); HIV diagnosis timepoint (n=9), CD4 count (n=23), VL (n=92 at 6 months, n=86 at 24 months); ART initiation (n=5), ART regimen (n=8 at 6 months, n=7 at 24 months); infant prophylaxis (n=4 at 6 months, n=2 at 24 months).
All HIV-related variables are cited out of the number of HIV-infected mothers with available data. Percentages are cited among those with non-missing values.

a) Maternal smoking was measured by urine cotinine levels taken antenatally/birth urine using the IMMULITE® 1000 Nicotine Metabolite Kit (Siemens Medical Solutions Diagnostics®, Glyn Rhonwy, United Kingdom). Levels ≥500 ng/ml quantified active smoking, 10-500 mg/ml as passive smoking and <10ng/ml as non-smoking.
b) Maternal alcohol use assessed and quantified using the Alcohol, Smoking and Substance Involvement Screening Test (ASSIST) and retrospectively collected data on moderate-severe alcohol use in pregnancy forming a dichotomous measure.
c) Maternal depression in pregnancy measured using the Edinburgh postnatal depression scale (EPDS), a threshold of >=13 was used as the threshold for depression.
d) The lowest maternal CD4 within one year prior to birth and 3 months post-birth was used to reflect maternal immunosuppression in pregnancy and maximise sample numbers.
e) Maternal viral load determined as the highest viral load during pregnancy Where there was more than one result, the highest viral load during pregnancy was taken and <40 copies/ml was classified as lower than the detectable limit, >=40-<1000copies/ml as detectable and >=1000copies/ml as unsuppressed.

Appendix 3: Mean BSID-III scaled scores by domain according to HIV exposure at 6 and 24 months

BSID-III Domain: Scaled scores	6 months			24 months		
	Mean (SD)		P	Mean (SD)		P
	HIV-exposed uninfected	HIV-unexposed		HIV-exposed uninfected	HIV-unexposed	
Cognitive	10·73 (2·46)	10·18 (2·59)	0·14	6·80 (1·88)	7·14 (1·84)	0·039*
Receptive language	10·38 (2·75)	9·96 (2·78)	0·31	6·62 (1·82)	7·25 (1·97)	0·0002*
Expressive language	10·95 (3·56)	11·18 (3·33)	0·65	6·94 (2·29)	7·57 (2·30)	0·0023*
Fine Motor	13·15 (2·95)	12·76 (3·18)	0·40	9·25 (2·62)	9·29 (2·45)	0·83
Gross Motor	11·00 (2·59)	10·61 (2·93)	0·35	8·14 (2·15)	8·34 (2·40)	0·34

Footnote:
*P<0·05. Significance values are from unpaired t-tests. Scaled scores are standardised to a reference population and have a mean of 10 and SD of 3. Scores at 6 months are corrected for prematurity.

At 6 months, the mean scaled scores of all subscales were within the BSID-III reference range (mean 10, SD 3). At 24 months, HEU children had mean scaled scores <-1 SD below the reference mean in cognitive, receptive language and expressive language but not in fine or gross motor.

Appendix 4: Child developmental outcomes at 24 months according to HIV exposure assessing the effect of breastfeeding on the exposure-outcome relationship.

We assessed duration of exclusive breastfeeding and exclusive breastfeeding to 6 months (defined as the proportion of infants aged over 5 months fed exclusively with breastmilk). These breastfeeding variables were separately included in the adjusted models to examine any impact on the association between maternal HIV and child development. The adjusted differences and odds ratios in the table relate to the child developmental outcomes according to HIV exposure.

Raw scores						
**BSID-III Domain**	**Original multivariable model**		**Adjusted for exclusive breastfeeding duration**		**Adjusted for exclusive breastfeeding to 6 months**	
	**Adjusted Difference (95% CI)****	**P**	**Adjusted difference (95% CI)****	**P**	**Adjusted difference (95% CI)****	**P**
Cognitive	-0·45 (-1·32 to 0·43)	0·32	-0·46 (-1·35 to 0·43)	0·32	-0·45 (-1·33 to 0·42)	0·31
Receptive language	-1·03 (-1·69 to -0·37)	0·0024*	-0·92 (-1·60 to -0·24)	0·0077*	-1·00 (-1·67 to -0·34)	0·0032*
Expressive language	-1·17 (-2·09 to -0·24)	0·013*	-1·10 (-2·05 to -0·15)	0·023*	-1·16 (-2·09 to -0·23)	0·015*
Fine Motor	0·09 (-0·49 to 0·66)	0·77	0·06 (-0·52 to 0·65)	0·83	0·07 (-0·50 to 0·65)	0·80
Gross Motor	-0·41(-1·09 to 0·27)	0·24	-0·46 (-1·15 to 0·23)	0·19	-0·40 (-1·09 to 0·28)	0·25

Delayed development (<-2SD)						
**BSID-III Domain**	**Original multivariable model**		**Adjusted for exclusive breastfeeding duration**		**Adjusted for exclusive breastfeeding to 6 months**	
	**Adjusted Odds Ratio (95% CI)****	**P**	**Adjusted Odds Ratio (95% CI)****	**P**	**Adjusted Odds Ratio (95% CI)****	**P**
Cognitive	1·01 (0·55 to 1·85)	0·97	0·93 (0·50 to 1·74)	0·82	1·00 (0·55 to 1·84)	0·99
Receptive language	1·96 (1·09 to 3·52)	0·025*	1·94 (1·07 to 3·54)	0·030*	2·00 (1·11 to 3·60)	0·021*
Expressive language	2·14 (1·11 to 4·15)	0·024*	1·95 (0·99 to 3·86)	0·055	2·15 (1·11 to 4·17)	0·024*
Fine Motor	1·53 (0·53 to 4·42)	0·44	1·53 (0·52 to 4·53)	0·44	1·52 (0·52 to 4·43)	0·44
Gross Motor	1·23 (0·44 to 3·43)	0·69	1·18 (0·41 to 3·35)	0·76	1·23 (0·44 to 3·42)	0·70

Footnotes:

*p<0·05; For the linear regression models negative estimates indicate maternal HIV status is associated with lower total raw scores in that domain, and therefore poorer outcomes.

For the logistic regression models odds ratios >1 indicate maternal HIV status is associated with higher risk of delay in that domain, and therefore poorer outcomes.

**Adjusting for child age and child sex, maternal education, household income, maternal age and breastfeeding variables where indicated.

Appendix 5: Child developmental outcomes at 24 months according to HIV exposure assessing the effect of prematurity (<37 weeks) on the exposure-outcome relationship.

BSID-III Domain	Raw Scores		Delay (<-2SD)	
	Adjusted difference (95% CI)**	P	Adjusted Odds ratio (95% CI)**	P
Cognitive	-0·46 (-1·33 to 0·40)	0·29	1·02 (0·56 to 1·88)	0·94
Receptive language	-1·04 (-1·70 to -0·38)	0·0021*	1·96 (1·09 to 3·53)	0·024*
Expressive language	-1·18 (-2·10 to -0·25)	0·013*	2·16 (1·11 to 4·20)	0·023*
Fine Motor	0·08 (-0·49 to 0·65)	0·78	1·53 (0·53 to 4·43)	0·43
Gross Motor	-0·41 (-1·08 to 0·27)	0·24	1·21 (0·44 to 3·39)	0·71

Footnotes:

Similar results are obtained if low birth weight [<2·5kg] used in place of prematurity.

*p<0·05; For the linear regression models negative estimates indicate HIV exposure is associated with lower total raw scores in that domain, and therefore poorer outcomes. For the logistic regression models odds ratios >1 indicate maternal HIV status is associated with higher risk of delay in that domain, and therefore poorer outcomes.

**Adjusting for child age and child sex, maternal education, household income, maternal age and prematurity

Appendix 6: Child developmental outcomes at 24 months according to HIV exposure assessing the effect of maternal depression on the exposure-outcome relationship.

BSID-III Domain	Raw Scores		Delay (<-2SD)	
	Adjusted difference (95% CI)**	P	Adjusted Odds ratio (95% CI)**	P
Cognitive	-0·54 (-1·47 to 0·39)	0·26	1·04 (0·54 to 1·98)	0·91
Receptive language	-0·96 (-1·67 to -0·25)	0·0084*	1·78 (0·96 to 3·31)	0·066
Expressive language	-1·20 (-2·18 to -0·22)	0·016*	2·06 (1·01 to 4·23)	0·048*
Fine Motor	-0·02 (-0·64 to 0·59)	0·94	1·56 (0·49 to 4·93)	0·45
Gross Motor	-0·56 (-1·30 to 0·18)	0·14	1·27 (0·45 to 3·63)	0·65

Footnotes:

Antenatal maternal depression was measured using the Edinburgh Postnatal Depression Scale (EPDS) where a cut-off score of >=13 was used as the threshold for depression. Depression was used as a proxy for maternal psychosocial illness as per the DAG, however, further work needs to be done to assess this potential mediator.

*p<0·05; For the linear regression models negative estimates indicate HIV exposure is associated with lower total raw scores in that domain, and therefore poorer outcomes. For the logistic regression models odds ratios >1 indicate maternal HIV status is associated with higher risk of delay in that domain, and therefore poorer outcomes.

**Adjusting for child age and child sex, maternal education, household income, maternal age and maternal depression as measured by the Edinburgh Postnatal Depression Scale.

Appendix 7: Restricted analysis of the site with the majority of HIV-exposed uninfected children, where isiXhosa was the home language (n=388, HIV-exposed uninfected 155, HIV-unexposed 233). Unadjusted and adjusted BSID-III domain scores at 24 months according to HIV exposure.

Raw scores						
**BSID-III Domain**	**Mean (SD)**		**Unadjusted Difference (95% CI)**	**P**	**Adjusted Difference (95% CI)****	**P**
	**HIV-exposed uninfected**	**HIV-unexposed**				
Cognitive	54·77 (5·04)	55·55 (4·79)	-0·79 (-1·79 to 0·21)	0·12	-0·43 (-1·51 to 0·65)	0·43
Receptive language	19·79 (3·53)	20·86 (3·47)	-1·07 (-1·79 to -0·36)	0·0034*	-0·81 (-1·57 to -0·05)	0·037*
Expressive language	22·79 (5·39)	23·95 (4·60)	-1·16 (-2·19 to -0·13)	0·028*	-0·98 (-2·09 to 0·13)	0·082
Fine Motor	37·32 (3·33)	37·48 (3·22)	-0·16 (-0·83 to 0·51)	0·63	0·13 (-0·59 to 0·86)	0·72
Gross Motor	53·07 (3·41)	53·02 (4·03)	0·05 (-0·74 to 0·85)	0·90	-0·16 (-1·02 to 0·70)	0·71
**Delayed development (<-2SD)**						
**BSID-III Domain**	**N (%) with delay**		**Unadjusted OR (95% CI)**	**P**	**Adjusted OR (95% CI)****	**P**
	**HIV-exposed uninfected**	**HIV-unexposed**				
Cognitive	17 (11%)	22 (10%)	1·17 (0·60 to 2·28)	0·65	1·0 (0·48 to 2·07)	0.99
Receptive language	21 (14%)	15 (7%)	2·27 (1·13 to 4·56)	0·021*	1·96 (0·92 to 4·16)	0·081
Expressive language	17 (12%)	15 (7%)	1·84 (0·89 to 3·82)	0·099	2·35 (1·04 to 5·32)	0·041*
Fine motor	6 (4%)	7 (3%)	1·30 (0·43 to 3·94)	0·65	1·11 (0·33 to 3·77)	0·86
Gross motor	6 (4%)	11 (5%)	0·81 (0·29 to 2·25)	0·69	0·98 (0·31 to 3·14)	0·97

Footnotes:

The Afrikaans-speaking site had lower HEU numbers and therefore limited power to detect a difference.
*p<0·05; For the linear regression models negative estimates indicate HIV exposure is associated with lower total raw scores in that domain, and therefore poorer outcomes. For the logistic regression models odds ratios >1 indicate maternal HIV status is associated with higher risk of delay in that domain, and therefore poorer outcomes.
**Adjusting for child age and child sex, maternal education, household income and maternal age.

Appendix 8: Analysis to assess the potential effect of alcohol exposure in pregnancy on the exposure-outcome relationship.

This analysis was performed due to the difference seen between those children with and without a BSID-III at 24 months, as more children with a BSID-III performed were exposed to alcohol in pregnancy than those without.

BSID-III Domain	Raw Scores		Delay (<-2SD)	
	Adjusted Difference (95% CI)**	P	Adjusted Odds ratio (95% CI)**	P
Cognitive	-0·53 (-1·46 to 0·41)	0·27	1·08 (0·57 to 2·07)	0·81
Receptive language	-0·95 (-1·67 to -0·24)	0·0093*	1·85 (1·00 to 3·44)	0·052
Expressive language	-1·24 (-2·22 to -0·25)	0·014*	2·17 (1·06 to 4·48)	0·035*
Fine Motor	-0·03 (-0·65 to 0·59)	0·92	1·50 (0·47 to 4·78)	0·50
Gross Motor	-0·57 (-1·31 to 0·18)	0·13	1·28 (0·46 to 3·61)	0·64

Footnotes:

The same result was seen if the analysis was stratified by alcohol exposure.

*p<0·05; For the linear regression models negative estimates indicate HIV exposure is associated with lower total raw scores in that domain, and therefore poorer outcomes. For the logistic regression models odds ratios >1 indicate maternal HIV status is associated with higher risk of delay in that domain, and therefore poorer outcomes.

**Adjusting for child age and child sex, maternal education, household income, maternal age and alcohol exposure in pregnancy.

Appendix 9: Restricted analysis of those HIV-exposed uninfected children born to mothers on first line ART (n=132). Unadjusted and adjusted BSID-III domain scores according to HIV exposure.

Raw scores						
**BSID-III Domain**	**Mean (SD)**		**Unadjusted Difference (95% CI)**	**P**	**Adjusted Difference (95% CI)****	**P**
	**HIV-exposed uninfected**	**HIV-unexposed**				
Cognitive	55·18 (5·05)	55·69 (4·73)	-0·51 (-1·42 to 0·40)	0·27	-0·19 (-1·15 to 0·77)	0·70
Receptive language	20·00 (3·46)	21·10 (3·72)	-1·10 (-1·80 to -0·40)	0·0022*	-0·88 (-1·61 to -0·15)	0·019*
Expressive language	22·97 (5·58)	24·45 (4·94)	-1·48 (-2·47 to -0·49)	0·0035*	-1·12 (-2·15 to -0·10)	0·032*
Fine Motor	37·50 (3·28)	37·51 (3·10)	-0·01 (-0·61 to 0·58)	0·96	0·16 (-0·47 to 0·79)	0·61
Gross Motor	53·34 (3·22)	53·31 (3·66)	0·03 (-0·67 to 0·73)	0·94	-0·16 (-0·91 to 0·59)	0·68
**Delayed development (< -2SD)**						
**BSID-III Domain**	**N (%) with delay**		**Unadjusted OR (95% CI)**	**P**	**Adjusted OR (95% CI)****	**P**
	**HIV-exposed uninfected**	**HIV-unexposed**				
Cognitive	13 (10%)	52 (9%)	1·08 (0·57 to 2·05)	0·81	0·98 (0·49 to 1·96)	0·96
Receptive language	17 (13%)	40 (7%)	1·94 (1·06 to 3·55)	0·031*	1·93 (1·00 to 3·72)	0·049*
Expressive language	14 (11%)	31 (6%)	2·10 (1·08 to 4·07)	0·029*	2·41 (1·16 to 5·03)	0·019*
Fine motor	5 (4%)	12 (2%)	1·82 (0·63 to 5·26)	0·27	1·67 (0·53 to 5·25)	0·38
Gross motor	3 (2%)	19 (4%)	0·68 (0·20 to 2·33)	0·54	0·79 (0·21 to 3·00)	0·72

Footnotes:

*p<0·05; For the linear regression models negative estimates indicate maternal HIV status is associated with lower total raw scores in that domain, and therefore poorer outcomes. For the logistic regression models odds ratios >1 indicate maternal HIV status is associated with higher risk of delay in that domain, and therefore poorer outcomes.

**Adjusting for child age and child sex, maternal education, household income and maternal age at delivery.
